# Anti-Quorum Sensing Activity of the Traditional Chinese Herb, *Phyllanthus amarus*

**DOI:** 10.3390/s131114558

**Published:** 2013-10-28

**Authors:** Kumutha Priya, Wai-Fong Yin, Kok-Gan Chan

**Affiliations:** Division of Genetics and Molecular Biology, Institute of Biological Sciences, Faculty of Science, University of Malaya, Kuala Lumpur 50603, Malaysia; E-Mails: legolaz_kp23@hotmail.com (K.P.); yfong@um.edu.my (W.-F.Y.)

**Keywords:** anti-pathogenic, bioluminescence, *Chromobacterium violaceum* CVO26, *N*-acyl-L-homoserine lactones (AHLs), *Phyllanthus amarus*, *Pseudomonas aeruginosa* PAO1, quorum sensing, pyocyanin, swarming, virulence factors

## Abstract

The discovery of quorum sensing in Proteobacteria and its function in regulating virulence determinants makes it an attractive alternative towards attenuation of bacterial pathogens. In this study, crude extracts of *Phyllanthus amarus* Schumach. & Thonn, a traditional Chinese herb, were screened for their anti-quorum sensing properties through a series of bioassays. Only the methanolic extract of *P. amarus* exhibited anti-quorum sensing activity, whereby it interrupted the ability of *Chromobacterium violaceum* CVO26 to response towards exogenously supplied *N*-hexanoylhomoserine lactone and the extract reduced bioluminescence in *E. coli* [pSB401] and *E. coli* [pSB1075]. In addition to this, methanolic extract of *P. amarus* significantly inhibited selected quorum sensing-regulated virulence determinants of *Pseudomonas aeruginosa* PA01. Increasing concentrations of the methanolic extracts of *P. amarus* reduced swarming motility, pyocyanin production and *P. aeruginosa* PA01 *lecA∷lux* expression. Our data suggest that *P. amarus* could be useful for attenuating pathogens and hence, more local traditional herbs should be screened for its anti-quorum sensing properties as their active compounds may serve as promising anti-pathogenic drugs.

## 1. Introduction

The emergence of more multidrug resistant pathogens at an alarming rate is now a major concern to the public health care and policy makers in the medical industry. The continuous misuse of broad-spectrum antibiotics has caused bacteria to develop resistance hence leading towards a continuous decline in the availability of antibiotics that are able to work against these pathogens. Quorum sensing (QS) is a cell-cell signaling system whereby bacteria produce signalling molecules, termed autoinducers, that coordinate the production of cell density-dependent regulatory factors as well as the expression of virulence factors in pathogens [1,2]. Thus, targeting the QS system to attenuate bacterial virulence and pathogenicity is a promising alternative without the use of antibiotic. Proteobacteria primarily utilize the LuxI/LuxR type QS-signalling system where *N*-acyl-homoserine lactones (AHLs) are the signalling molecules whereas post-translationally modified peptides regulate QS systems in Gram-positive bacteria [3].

One of the best characterised QS models is *Pseudomonas aeruginosa*, which consists of two QS systems namely *lasI*/*R* and *rhl*/*R*, which function in a hierarchal way, and regulate expression of several virulence genes [3–5]. Cytotoxic lectins, elastases, pyocyanin, biofilm formation, rhamnolipid synthesis, and hydrogen cyanide synthase are among the QS-mediated virulence factors in *P. aeruginosa* [6–9].

Anti-QS compounds may target the QS-systems either by disrupting the AHL synthase, the signal molecule itself, or AHL target receptors [10]. Among the plant-derived sources with anti-QS properties that have been previously studied are Malabaricone C (from *Myristica cinnamomea)* [11], caffeine [12], *Melicope lunu-ankenda* [13], *Syzygium Aromaticum* [14], and *Terminalia catappa* [15].

**Figure 1.**
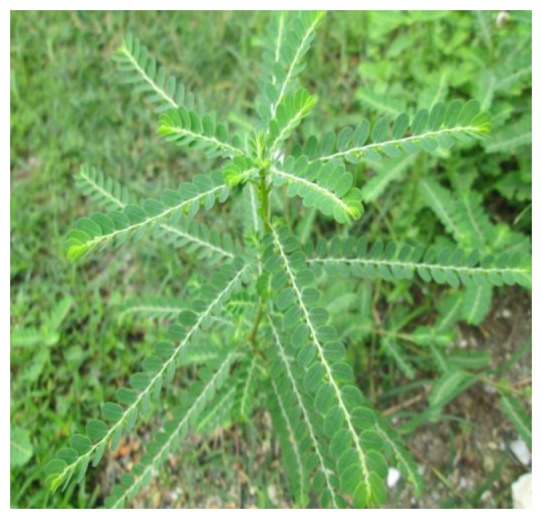
*Phyllanthus amarus* Schumach. & Thonn.

*Phyllanthus amarus* Schumach. & Thonn (Figure 1) is a small woody shrub most commonly found growing in uncultivated or wastelands in tropical regions. *P. amarus* has been tested and shown to be anti-diabetic [16], hepatoprotective [17], anti-inflammatory [18] and chemoprotective [19], along with many other medicinal properties, but its anti-QS properties had not been tested. In this study, we report for the first time the anti-QS properties of *P. amarus* and its methanolic crude extract is shown to inhibit selected virulence factors of *P. aeruginosa* PAO1.

## 2. Experimental Section

### 2.1. Identification of Plant Sample, Deposition of Voucher Specimen, and Extract Preparations

*P. amarus* samples were collected from wastelands around the grounds of University Malaya. Voucher specimens of *P. amarus* Schumach. & Thonn. (family: Phyllanthaceae) (KLU 47768) were deposited in the Herbarium of University of Malaya. Collected samples were washed with sterile distilled water and rinsed with 70% (v/v) ethanol prior to drying in a 40 °C oven for three days. Dried samples were ground using an industrial grade blender. Powdered plant samples were treated sequentially with organic solvents (hexane, chloroform and methanol) of increasing polarity (in a ratio of 1:10 w/v). Samples were soaked in each solvent for three days with shaking at 220 rpm. The extracts of each solvent were filtered using Whatman No.1 filter paper. This step was repeated twice to ensure all of the fine powder was completely removed before proceeding to the removal of solvents using a rotary evaporator (EYELA, Tokyo, Japan). The crude extracts were then dried in a fume hood and later placed in a desiccator. Stock solutions of 10 mg/mL were made using 100% DMSO (Merck KGaA, Darmstadt, Germany) and later diluted to (in mg/mL) 1, 2, 3, 4 and 5 using ultrapure water (Mili-Q, Merck KGaA, Darmstadt, Germany) prior to use. The remaining crude extracts were stored at −20 °C.

### 2.2. Biosensors and Culture Conditions

The biosensor strains were routinely cultured in Luria-Bertani medium (Scharlab, Barcelona, Spain). *Chromobacterium violaceum* CV026 was cultured at 28 °C whilst *P. aeruginosa* and *Escherichia coli* lux biosensor strains were cultured at 37 °C. The growth medium for *E. coli* [pSB401] and *E. coli* [pSB1075] were supplemented with 20 μg/mL tetracycline. *C. violaceum* CV026 used in this study is a double mini-Tn5 mutant derived from ATCC 31532, Kan^R^, Hg^R^, cvil∷Tn5xylE, and a spontaneous Str^R^. It produces a purple pigment, namely violacein, in the presence of exogenously supplied short chain AHLs [20]. *E. coli* [pSB401] was constructed using luxR/luxl' (*Photobacterium fisheri* [ATCC 29999]) fusion; pACYC184-derived, Tet^R^ whereas *E. coli* [pSB1075] was a result of lasR/lasl (*P. aeruginosa* PAO1)∷luxCDABE (*Photorhabdus luminescens* [ATCC 29999]) fusion in pUC18 Tet^R^. Both these AHL biosensors produce bioluminescence in the presence of exogenously supplied AHLs [21].

### 2.3. Bacterial Growth

To rule out the possibility of the plant extracts having antibacterial activity against the biosensor cells that might lead to a false anti-QS result, we first determined the antibacterial activity using a previously reported method [22] with some modifications. Briefly, the optical density at 600 nm (OD_600_) of overnight grown cultures of *C. violaceum* CV026, *P. aeruginosa* PAO1, *E. coli* [pSB401] and *E. coli* [pSB1075] were adjusted to OD_600_ of 0.1. Next, 230 μL of the bacterial culture and 20 μL of the plant extracts were placed in each well of the 96-well microtitre plate. The microtitre plates were incubated at the optimum temperature of the tested bacteria and the OD_600_ were measured every 30 min for 24 h by using Tecan luminometer (Infinite M200, Mannerdorf, Switzerland).

### 2.4. QS Inhibition

Bioassay was performed as described by Tan *et al.* [13] with several modifications. Briefly, overnight CV026 culture (15 mL) was seeded into warm molten LB agar (200 mL) and supplemented with C6-HSL to a final concentration of 0.25 mg/mL. The mixture was swirled gently to ensure complete mixing before being poured into Petri dishes. Wells were made using sterile 1 μL white pipette tips. Next, 20 μL of plant extracts of various concentrations (1–5 mg/mL) were loaded in the wells and DMSO (10%–50% (v/v)) served as negative controls that corresponded to each of the plant extracts concentration used in the bioassay. The plates were then incubated overnight in an upright position at 28 °C. A ring of colourless and turbid (halo zone) growth on a purple background indicated anti-QS properties of the extracts [23].

### 2.5. Quantification of Bioluminescence

Bioluminescence was quantified using biosensor strains *E. coli* [pSB401] and *E. coli* [pSB1075], and inhibition of *P. aeruginosa* PAO1 lecA expression was performed using *P. aeruginosa* PAO1 lecA∷lux. Luminescence from these biosensors were performed as previously described [24]. Overnight cultures of *E. coli* [pSB401], *E. coli* [pSB1075] and *P. aeruginosa* PAO1 lecA∷lux were diluted to OD_600_ of 0.1. Cultures of *E. coli* [pSB401] and *E. coli* [pSB1075] were supplemented with 0.00005 μg/mL [N-[3-oxo-hexanoyl]-L-homoserine lactone [3-oxo-C6-HSL] and 0.5 μg/mL N-[3-oxo-dodecanoyl] homoserine lactone [3-oxo-C12-HSL], respectively. Aliquots of bacterial culture (230 μL) were loaded into each well containing 20 μL of plant extract. Luminescence and bacterial growth (OD at 495 nm) were measured every 30 min for 24 h using a Tecan Infinite M200 luminometer. Measurement of luminescence is expressed as relative light units (RLU) per OD (at 495 nm).

### 2.6. Attenuation of P. aeruginosa PAO1 Virulence Factors

#### 2.6.1. Pyocyanin Quantification Assay

Pyocyanin from *P. aeruginosa* PAO1 was quantified using methods as described previously [13,14,25]. Briefly, overnight grown culture of *P. aeruginosa* PAO1 were adjusted to OD_600_ of 0.1. Next, plant extract (250 μL) was mixed well with PAO1 cultures (4.75 mL) and incubated at 37 °C for 18 h. To extract pyocyanin from the cultures (5 mL), the bacterial cultures were extracted with chloroform (3 mL) and vortexed vigorously. The chloroform layer was then transferred to a new, sterile polypropylene tube and re-extracted with 1 mL HCl (0.2 M). After centrifugation, the top layer (HCl) was removed and the absorbance was measured at 520 nm using UV-visible spectrophotometer (Cary 60 UV-Vis Spectrophotometer, Agilent, Santa Clara, CA, USA). DMSO of 10%–30% (v/v) were used as negative controls.

#### 2.6.2. Swarming Assay

*P. aeruginosa* PAO1 swarming plates were prepared as follows: glucose (1% w/v), Bacto agar (0.5% w/v), Bacto peptone (0.5% w/v), and yeast extract (0.2% w/v) as described previously [14]. An overlay of 10 mL swarming agar was made prior to adding an additional layer of 5 mL swarming agar seeded with 250 μL of plant extract. Once the agar had solidified, 2 μL of overnight *P. aeruginosa* PAO1 was inoculated in the middle of the agar and incubated at 37 °C for 16 h. Impaired swarming motility of *P. aeruginosa* PAO1 indicates anti-QS properties of the plant extract.

### 2.7. Statistical Analysis

All the results represented were means ± standard deviations of three independent experiments carried out in triplicates. The significance of all the data was tested using Student's t-test (*p* < 0.05) using the GraphPad Prism software.

## 3. Results and Discussion

### 3.1. Screening of Plant Extracts Anti-QS Properties Using Chromobacterium Violaceum CV026

*C. violaceum* CV026 is a double Tn*5* mutant that is only able to produce the purple pigment violacein in the presence of an exogenously supplied short chain AHL [20]. In this study, only the methanolic extract of *P. amarus* exhibited a notable halo zone on the purple CV026 lawn.

**Figure 2.**
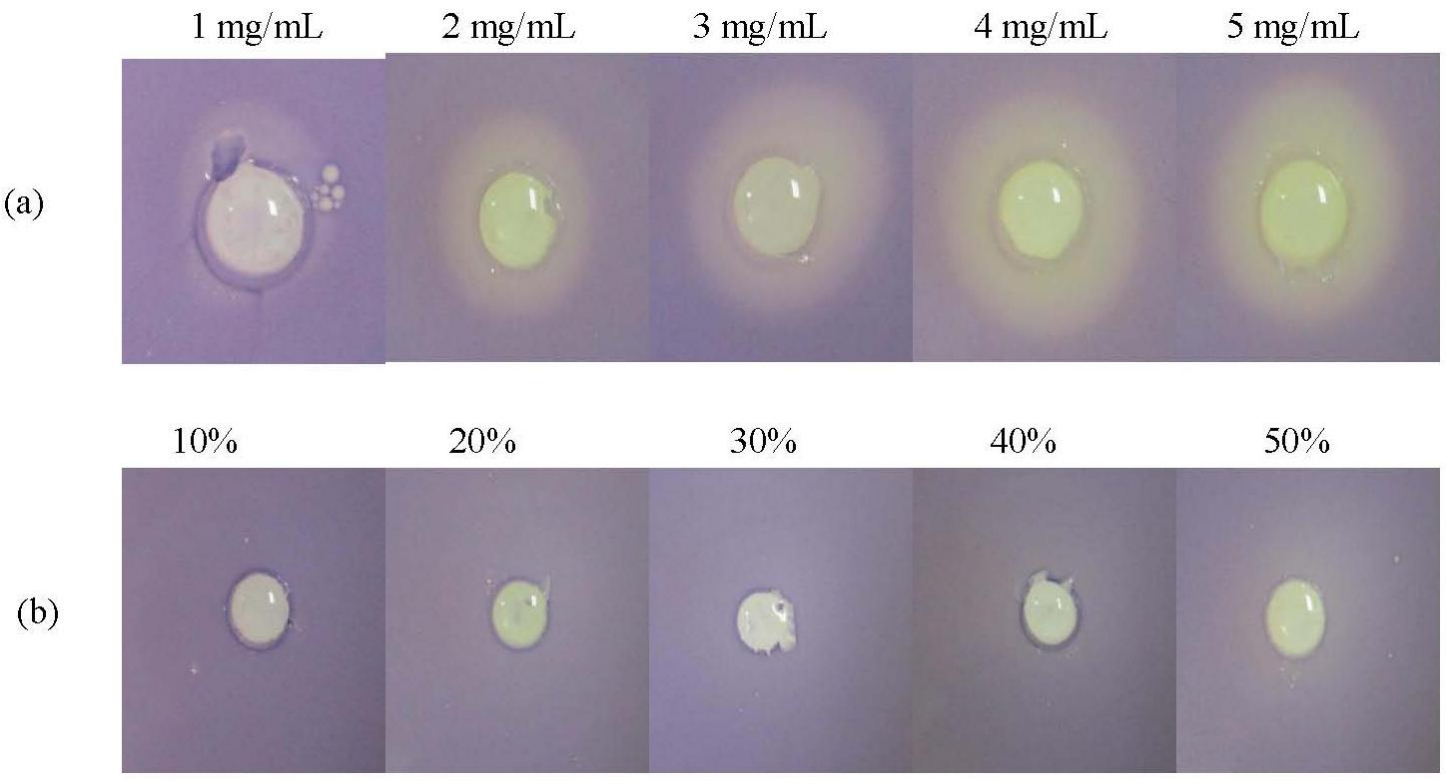
Figure below shows QS-inhibition of methanolic extract at 1, 2, 3, 4, and 5 mg/mL (**a**) and the corresponding negative control (**b**) DMSO at 10%–50% (v/v).

Hexane and chloroform extracts of *P. amarus* did not cause any observable halo zone (data not shown). Figure 2 shows that the formation of halo zone was observable at 1 mg/mL and increased in diameter as the concentration increased to 5 mg/mL. On the other hand, DMSO (negative control) did not show any bactericidal or anti-QS effect. Therefore, formation of halo zone indicated anti-QS activity of the methanolic extract.

### 3.2. Bacterial Growth

**Figure 3.**
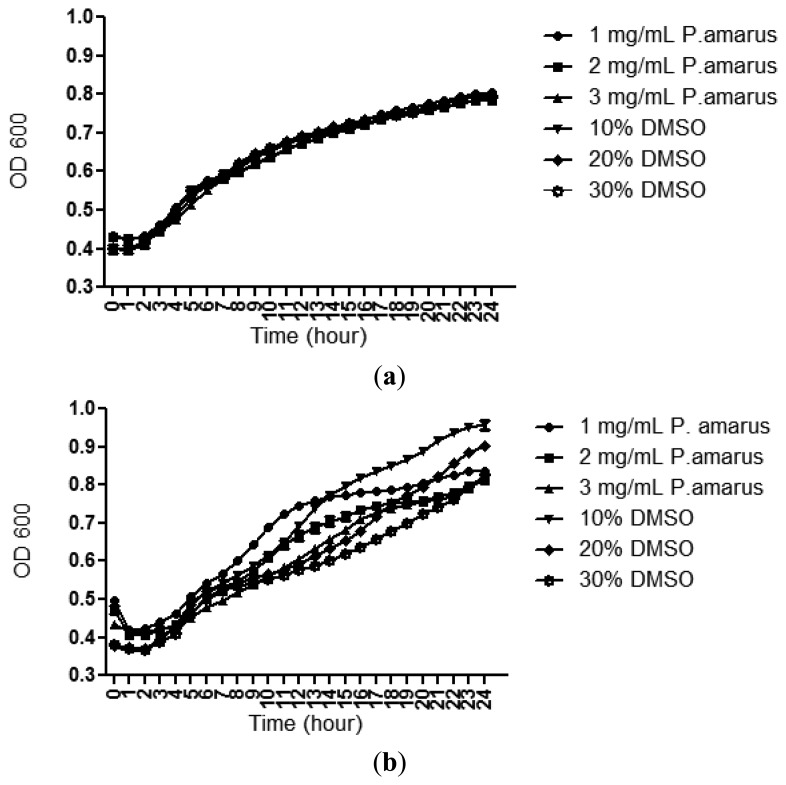
Growth of *C. violaceum* (**a**) and *P. aeruginosa* (**b**) supplemented with 1 to 3 mg/mL methanolic extract of *P. amarus*.

A bacterial growth curve (OD_600_) was determined over a period of 24 h to rule out any antibacterial properties of the methanolic extracts of *P. amarus* that may inhibit growth of *C. violaceum* CV026 and *P. aeruginosa* PAO1. Figure 3 confirms that the methanolic extract did not show inhibition against the growth of both the bacterial strains used in this study although this plant has been shown to exhibit antibacterial activity by previously reported work [26].

### 3.3. Quantification of Bioluminescence

#### 3.3.1. Methanolic Extract of *P. amarus* Inhibited lux-Based Biosensors

**Figure 4.**
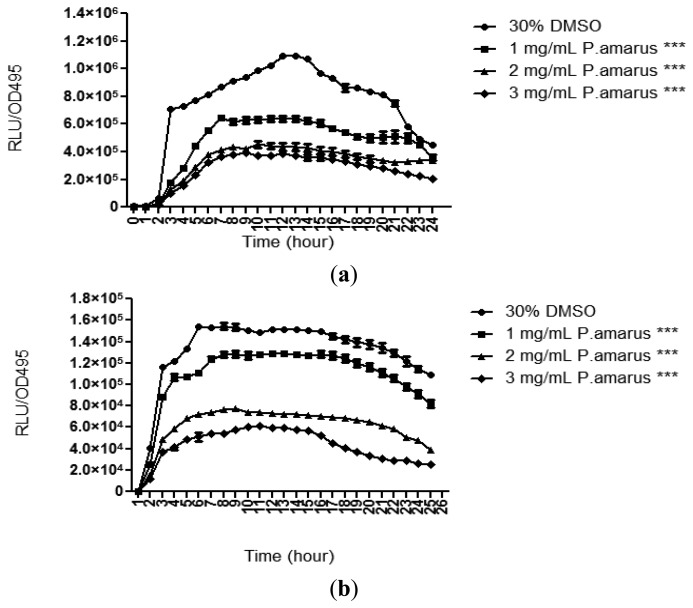
The methanolic extract of *P. amarus* reduced bioluminescence produced by (**a**) *E. coli* [pSB401] and (**b**) *E. coli* [pSB1075]. Statistical analysis was done using Student's t-test with *p*-value < 0.05 being very significant (noted with ***).

Methanolic extract of *P. amarus* was further tested for its anti-QS properties against both long and short chain AHLs using lux-based biosensors, namely *E. coli* [pSB401] and *E. coli* [pSB1075], which respond to short and long chain AHLs, respectively, by producing bioluminescence [21]. The methanolic extract of the plant significantly reduced the luminescence produced by both the biosensor strains with increasing concentration as compared to DMSO (30% v/v, negative control). The methanolic extract of *P. amarus* was more effective at reducing the luminescence produced by *E. coli* [pSB401] as the inhibition was observable at the lowest concentration of 1 mg/mL (Figure 4). As for *E. coli* [pSB1075], luminescence was greatly reduced starting at 3 mg/mL.

#### 3.3.2. Inhibition of *P. aeruginosa* PAO1 lecA Expression

**Figure 5.**
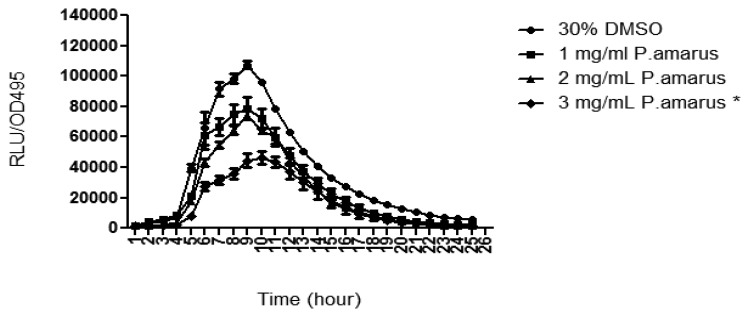
The inhibition of *P. aeruginosa* PAO1 *lecA* expression at concentration of 3 mg/mL. Statistical analysis was done using Student's t-test with *p*-value < 0.05 being significant (noted with *).

*P. aeruginosa* produces a variety of exoproducts to sustain its survivability and maintain its virulence. Among the exoproducts released are two types of soluble carbohydrate-binding cytotoxic protein termed lectins, LecA (PA-IL) and LecB (PA-IIL) [27]. LecA have been studied to have cytotoxic effect towards respiratory cells and playing a role in biofilm formation [28,29]. In order to study the lecA expression of *P. aeruginosa*, lecA∷luxCDABE reporter fusion was constructed by inserting luxCDABE from *Photorhabdus luminescens* into the lecA gene region of *P. aeruginosa* [6]. Figure 5 shows that the methanolic extract of *P. amarus* reduced PAO1 lecA expression with increasing concentration. However, inhibition of lecA expression was significant at 3 mg/mL indicating that higher concentration was required for inhibition.

According to Winzer and co-workers, lecA expression is tightly regulated by the RhlR/C4-HSL QS system. Furthermore, PAO1 mutant rpoS impaired lectin synthesis, adding that both RpoS and Rhl/C4-HSL are needed for LecA synthesis [6]. This indicates that the methanolic extract of *P. amarus* may have anti-QS effects on the RhlR/C4-HSL QS-system or on the expression of rpoS. However, more work needs to be done to confirm this hypothesis.

### 3.4. Inhibition of P. aeruginosa PAO1 Swarming and Pyocyanin Production

#### 3.4.1. Swarming Assay

**Figure 6.**
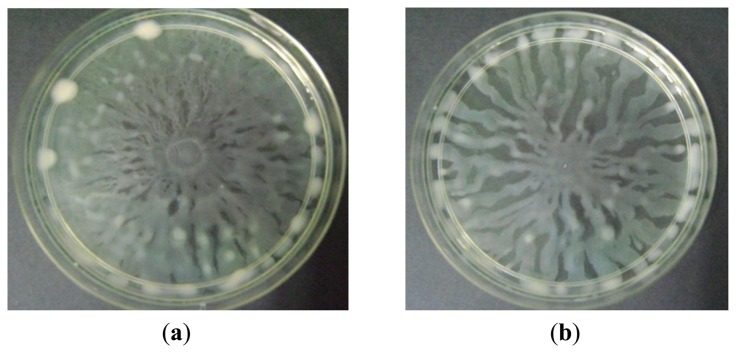

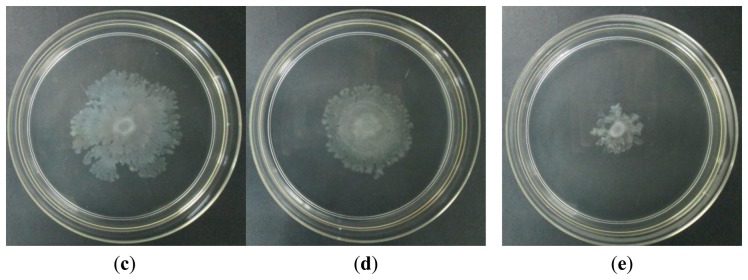
Swarming inhibition by increasing concentration of *P. amarus* methanolic extract. Swarming agar inoculated with (**a**) *P. aeruginosa* and supplemented with (**b**) 30% DMSO (v/v) and methanolic extracts of (**c**) 1 mg/mL, (**d**) 2 mg/mL, (**e**) 3 mg/mL.

Swarming is an uncoordinated surface-associated motility of hyperflagellated and highly motile bacterial cells which functions to colonise a niche [30,31]. In this assay, increasing concentration of methanolic extract of *P. amarus* inhibited swarming of *P. aeruginosa*. Swarming inhibition was observable at the lowest concentration of 1 mg/mL (Figure 6c). Swarming of *P. aeruginosa* was proven to be QS-system mediated whereby *lasI*/*lasR* mutants reduced and delayed swarming adding to *rhlI*/*rhlR* mutants that completely diminished swarming ability of the bacterium [32]. Rhamnolipid, a lipopeptide biosurfactant produced by *P. aeruginosa*, plays a crucial role in swarming motility and is QS-regulated [4,33]. Thus, compounds in the plant extract may have acted by interrupting the QS-systems or caused dysregulation during the synthesis of rhamnolipid. Based on earlier studies, swarming characteristics had been identified in other prokaryotes such as *Serratia liquefaciens*, *Escherichia coli*, and *Salmonella typhimurium* [34–36]. This suggests that swarming plays a role in bacterial colonisation in an environment or host [33].

#### 3.4.2. Pocyanin Assay

**Figure 7.**
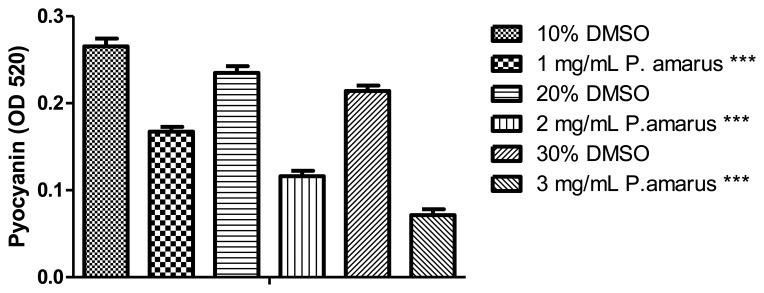
Methanolic extract of *P. amarus* inhibited *P. aeruginosa* PAO1 pyocyanin production. *** denoted statistical analysis performed using Student's t-test with *p*-value < 0.05 being significant.

Pyocyanin is a blue-redox reactive toxic exoproduct produced by *P. aeruginosa* [7]. Methanolic extract of *P. amarus* significantly reduced pyocyanin production with increasing concentration as compared to DMSO that served as negative control (Figure 7).

*P. aeruginosa* was found to be the predominant opportunistic pathogen that colonizes the airways of cystic fibrosis (CF) patients and a primary causative agent in sepsis of burned and immunocompromised patients [7]. Pyocyanin contributes to the persistence of *P. aeruginosa* infection by causing detrimental effects toward lung epithelial cells and by dysregulating inflammatory response initiated by the host [37,38]. Pyocyanin synthesis is regulated by a complex synchrony of *lasR-lasI, rhlR-rhlI*, and *mvfR-haq*QS-system whereby mutations in these systems led to the deficiency of pyocyanin synthesis [4,9,39]. Compounds in the extract may have inhibited the QS-systems at its gene expression level or act as an antagonist against AHL signalling molecules.

## 4. Conclusions

Based on the results obtained from this study, it is proven that the methanolic extract of *P. amarus* possesses anti-QS capabilities as well as being able to attenuate QS-regulated virulence determinants of *P. aeruginosa* POA1. Active compounds in the extract may provide a new insight towards discovering potential anti-pathogenic drugs to combat emerging multidrug resistant pathogens. Future work should direct to the isolation and characterisation of active molecule that is responsible for the anti-QS properties of the methanolic extract of *P. amarus*.
